# Variability in plant trace element uptake across different crops, soil contamination levels and soil properties in the Xinjiang Uygur Autonomous Region of northwest China

**DOI:** 10.1038/s41598-021-81764-w

**Published:** 2021-01-22

**Authors:** Weiguo Liu, Xiaodong Yang, Luchun Duan, Ravi Naidu, Kaihong Yan, Yanju Liu, Xiyuan Wang, Yongchao Gao, Yinguang Chen

**Affiliations:** 1 Institute of Resources and Environment Science, Xinjiang University, Urumqi, 830046 China; 2 Department of Geography and Spatial Information Technology, Ningbo University, NO.1188 North ring Road, Ningbo, 315211 China; 3 Global Centre for Environmental Remediation (GCER), The University of Newcastle (UON), Newcastle, NSW 2308 Australia; 4 Cooperative Research Centre for Contamination Assessment and Remediation of the Environment (CRC CARE), The University of Newcastle (UON), Newcastle, NSW 2308 Australia; 5 Ecology Institute, Shandong Provincial Key Laboratory of Applied Microbiology, Qilu University of Technology (Shandong Academy of Sciences), Jinan, 250306 China; 6 State Key Laboratory of Pollution Control and Resource Reuse, School of Environmental Science and Engineering, Tongji University, Shanghai, 200092 China

**Keywords:** Environmental chemistry, Environmental impact

## Abstract

This study investigated contamination status of eight trace elements (As, Cd, Cr, Hg, Pb, Cu, Zn and Ni) in farmland soils and crops at 535 sites across the Xinjiang Uygur Autonomous Region, Northwest China. Land use types of the sampling sites included vegetable patch, grain field and orchard. Our experimental results indicated all farmland soils were considered as trace element contamination based on the Nemerow comprehensive pollution index (*NCPI* > 1). However, 91.97% of the crop samples were uncontaminated according to the Chinese Risk Control Standard. Soils from the vegetable patch showed higher pollution level comparison with that from grain field and orchard. Health risks for both non-carcinogenic and carcinogenic risks were calculated through crop ingestion exposure pathway. Grain samples showed highest health risks, followed by melon and fruit, and vegetables. The health risks of crops were mainly driven by Cr and Cd. Crop consumption may pose risks for children but not adults. The source of trace element contamination in the different farmland soils varied and may be attributed to the different agricultural activities. Plant type had a greater influence on the trace element accumulation in crops compared with soil trace element contents and physicochemical properties.

## Introduction

Over the past 40 years, China has experienced remarkable industrialisation, along with use of large amounts of pesticides and fertilisers, which have resulted in considerable trace element pollution and soil degradation^1–4^. A recent national survey showed that 1/5 of China's farmland was contaminated by trace elements, with Cd, Ni, Hg and As being the major contaminants^5^. As trace element in soil can be accumulated by crops, there is a potential that they can enter food chain and accumulated in human body^1,6,7^. In fact, an increasing number of reports have highlighted the presence of trace elements in crops, grown in mining areas or farmlands nearby a point source such as an industrial or traffic zone, which have caused considerable health issues in humans^3,7–10^. Food safety problem associated with trace element contamination has become a prominent public concern in China, threatening the stability of society and the sustainability of the country’s economic growth^3,4,11^.

Agricultural activities are considered to be the major source for soil trace element pollution in farmland^12–14^. Trace element contamination status of soil and potential risks vary among different farmlands, such as grain fields, orchards and vegetable patches, due to the differences in agricultural activities (i.e., irrigation, fertilisation, pesticide spraying, mulching plastic film and tillage)^1,9,15^. As the majority of trace elements in crops are absorbed from soil via root uptake^14,16^, variations in agricultural activities may cause the difference in the contamination status and potential health risks of trace elements among different crops^3,15,17^. While trace element contamination in crops is more harmful to human health than that in soils^18,19^, the current research focus has been mainly on the influences of agricultural activities on the soil trace element contamination and the potential risks of different farmlands^1,11,13,20^, with very limited data relating to the edible parts of crops and the potential risks for human exposure^10,18,21^.

The potential risks of trace elements in crops may be affected by the their content in the soil or soil contamination status, soil physiochemical properties and plant species^18,19^. For example, soil pH has been recognised as an important parameter influencing trace element bioavailability due to the obvious relation with the activity and valence of trace elements^22,23^. Trace element ions are mainly absorbed on the hydroxyl surfaces of oxides or clay minerals. Soil pH would promote the adsorption/desorption of trace elements by regulating the quantity of hydrogen ions^23^. Higher soil organic matter (SOM) is conducive to reducing the soil trace element concentration due to complexation and adsorption^22,24^. Also, longer growth time may allow crops to absorb more trace elements from soil^25^. However, till now, the relative importance of these influencing factors on the trace element contents of crops are still few investigated^22,24,26,27^.

In this study, the content of seven trace metals (Cd, Cr, Hg, Pb, Cu, Zn and Ni) and one metalloid (As) in both the edible parts of three crops (grains, vegetables, melons and fruits) and the corresponding surface farmland soils (0–20 cm, in grain field, vegetable patch and orchard) were measured at 535 sampling points across the Xinjiang Uygur Autonomous Region (Xinjiang for short in the text below). The contamination status and health risks of trace elements based on potential crop consumption were assessed. The sources of the trace elements in the soil and the relative importance of influencing factors of the trace elements in crops were also analysed to achieve the following aims: (1) reveal how trace element contamination status and potential risks varies among different types of farmlands and corresponding crops; and (2) identify the potential influencing factors for trace element accumulation in the edible parts of crops.

## Materials and methods

### Study area

Xinjiang (E73°40′–96°18′, N34°25′–48°10′) is the largest provincial-level administrative region of China, located in northwest China, with an area of over 1.66 × 10^6^ km^2^. It has a typical continental climate, with an annual sunshine duration of 2600–3400 h, and the average monthly temperature ranges from − 20 °C to 33 °C. The average annual precipitation of this region is about 170 mm^28,29^. Xinjiang is the most important region for producing grain and fruits in China, with about 6.13 × 10^4^ km^2^ of farmland, accounting for 3.88% of the total area of China’s farmland, and has been planted with over 30 different kinds of crops^28^. The crops are classified into three types: grain, vegetables, melon and fruits. The Statistical Yearbook of Xinjiang (2015) has shown that the annual yields of these three crops are about 1.50 × 10^7^, 1.93 × 10^7^ and 0.96 × 10^7^ tons, respectively.

### Sample collection and analysis

The edible parts of the three types of crops (grain, melon and fruits, vegetables) and the surface soil (0–20 cm) near the crop’s roots were collected at 535 sites in Xinjiang (supporting information Fig. S1), and then transported to the Plant Physiology Laboratory of Xinjiang University during growth season from May to October, 2017. In total 63 samples were collected from grain fields, 337 from vegetable patches and 135 from orchards. The 535 sampling sites were not evenly distributed spatially due to farmland area and population distribution, which located in Urumqi (121), Changji (102), Turpan (45), Tacheng (18), Kumul (24), Altay (32), Bortala (16), Korla (104), Aksu (38) and Kashgar (35). No samples were collected in Hotan and Karamay regions because they only accounted for a small proportion of agricultural land of Xinjiang. In order to ensure the samples are representative of the typical farmland use of the region, each sampling site has to meet the selection criteria that it has been used to grow the same type of crops for more than three years continuously. The vegetables sampled include a total of 24 species while melons and fruits include 6 different species. Corn was chosen to represent grain as it has the largest planting area and widely distributed in Xinjiang. Other corn species such as sorghum, wheat, soybean and rice, however, are not the main grain crops in Xinjiang, hence only planted in a small range. Details of sampled crops are provided in supporting information Tables S1–S3.

All soil samples were air-dried and sieved to pass through a 0.15-mm mesh of a nylon screen. The samples were kept in two zip-lock plastic bags separately. One was used to determine the soil trace element contents and the other was used to measure pH, SOM and cation exchange capacity (CEC). Aqua regia (HCl:HNO_3_ = 3:1) was used to digest the soil samples, and the trace element contents were analysed by ICP-MS according to the reference (HJ 803-2016). Each sample was analysed in triplicates, and the mean value was reported as the final result. A set of geochemical soil standard reference samples (GSS Series, PMC Engineering, Danbury, CT, USA) was used for quality control. The recovery of trace elements was within the acceptable range of the test results’ quality control requirements set by National Standard Substances of China (95-105%). Soil pH was measured in soil :deionised water suspension (1:2.5) using a pH meter (PHS-25, Shanghai Electronics Science Instrument Co. Ltd., Shanghai, China). Soil CEC and SOM were measured using the NH_4_^+^ exchange method and the oil bath-K_2_CrO_4_ titration method, respectively^30,31^.

The edible parts of crops samples were firstly rinsed thoroughly with Milli-Q water and air-dried, followed by oven-dried at 80 °C to reach a constant weight. The dried samples were then ground into fine powders with pestle and mortar and stored in sealed plastic bags. The trace element contents in crops were determined followed Yang et al.^32^ and the guideline of trace element measurement in China (DB65/T 3971-2017), which is briefly described as follows. Each crop sample (0.05 g) was placed in a digestion tank. 2 mL H_2_O_2_ (analytical grade) and 2 mL HNO_3_ (analytical grade) were added. The digestion tank was then sealed up and placed into an oven heated at 180 °C for 10 h. After cooling down, 1% HNO_3_ solution was used to transfer each digestion solution into a 50 mL measuring flask. Estimation of all crop samples were conducted in triplicates, and the whole process was followed by blank samples.

Rhodium (^103^Rh) and Rhenium (^185^Re) standard solutions (1:1) were prepared into a mixed internal standard solution. 1% HNO_3_ solution was used to dilute digestion solution into a series of standard solutions with concentrations of 0, 0.5, 2.0, 5.0, 10.0 and 100.0 g/L for Pb, Cd, Cr, Hg, Cu and Ni, while of 0, 5, 10, 20, 50 and 100 g/L for Zn and As. The calibration of inductivity coupled plasma mass spectrometer (ICP-MS) was using a 10 g/L tuning fluid (including ^7^Li, ^59^Co, ^115^In and ^238^U). A vegetable standard reference sample (GBW 10014/GSB-5 cabbage, China) was used for quality control which were determined for the eight trace element contents. Pb and Ni were tested using in ordinary mode of ICP-MS, while Zn, Cd, Cr, As, Hg and Cu were tested by CCT mode. The detection limit of As, Cd, Cr, Cu, Hg, Ni, Pb and Zn were 0.01, 0.001, 0.20, 0.003, 0.05, 0.02 and 0.02 mg·kg^−1^ respectively. The recovery of trace elements was 94–104%, while RSD ranged from 0.01 to 5.01% for reference crops.

### Nemerow comprehensive pollution index calculation

The Nemerow comprehensive pollution index (*NCPI*) was calculated to evaluate the contamination status of trace elements in the soils^1,33^ (Eq. 1). Where *C*_*i*_ is the pollution value of the trace element in the sampling site *i*. *S*_*i*_ is the local background value of the target soil trace elements. The background values of the eight trace elements in Xinjiang are: As (11.20), Cd (0.12), Cr (39.6), Hg (0.02), Pb (13.5), Cu (35.8), Zn (76.8), Ni (26.4) (mg kg^−1^)^34^. The classification grades obtained using *NCPI* are shown in supporting information Table S4^33^.

1 $$ NCPI = \sqrt {\left( {\frac{{C_{i} }}{{S_{i} }}} \right)_{ave}^{2} + \left( {\frac{{C_{i} }}{{S_{i} }}} \right)_{max}^{2} } $$

### Health risk assessment of crop ingestion

The dietary consumption of crops containing trace elements is considered as the major source of hazardous element exposure in humans^10,20,33^. The average daily dose (*ADD*) (mg kg^−1^ day^−1^) of trace element via dietary consumption was assessed based on the U.S. Exposure Factors Handbook (Eq. 2)^35,36^. Where *C* is the average content of trace elements in the edible parts of crops (mg kg^−1^); *IngR* is the daily intake of the edible parts of crops (Adult: 450 mg·day^−1^; Child: 230 mg·day^−1^); *EF* and *ED* are the exposure frequency (365 days·year^−1^), and the average human exposure time (years, Adult: 24; Child: 6), respectively; *BW* and *AT* are the body weight (Adult: 53.1 kg; Child: 15 kg) and the average exposure time (Adult: 24 × 365 days; Child: 6 × 365 days), respectively. *AT* = *ED* × 365 (days) for a non-carcinogenic effect, and *AT* = 70 × 365 days for a carcinogenic effect were used.

2 $$ ADD = \frac{C \times IngR \times ED \times EF}{{BW \times AT}} \times 10^{ - 6} $$

The hazard quotient (*HQ*) is used to calculate the non-carcinogenic risk of residents consuming crops (Eq. 3). Where *R*_*f*_*D* is the estimated maximum permissible risk to humans through daily exposure: As (3.00 × 10^–4^), Cd (1.00 × 10^–3^), Cr (3.00 × 10^–3^), Hg (3.00 × 10^–4^), Pb (3.50 × 10^–3^), Cu (4.00 × 10^–2^), Zn (3.00 × 10^–1^) and Ni (2.00 × 10^–2^) (mg kg^−1^ day^−1^). *HQ* ≤ 1 indicates that potential non-carcinogenic effects are unlikely to occur, whereas *HQ* > 1 suggests their occurrence^36^.

3 $$ HQ = \frac{ADD}{{R_{f} D}} $$

For multiple trace elements, the hazard index (*HI*) is the sum of the *HQ* values of the individual element (Eq. 4). *HI* ≤ 1 and *HI* > 1 indicate potential risks do not occur and possibly occur, respectively. The greater *HI* indicates the higher probability of the occurrence of potential risks. *HI* > 10 indicates the occurrence of chronic toxic effects^35,37^. Based on previous studies, Cd, As, Cr and Pb are the most essential elements that causing potential risks, especially carcinogenic risk, thus *HI* is the sum of these four trace elements^1,9^.

4 $$ HI = \mathop \sum \limits_{i = 1}^{4} HQ_{i} $$

Carcinogenic risk can be evaluated by the following Eqs. (5) and (6)^1,37^. Where cancer risk (*CR*) and *ADD*_*i*_ are the probability of an individual developing cancer, and the daily intake dose of carcinogens (mg·kg^−1^·day^−1^), respectively; Slope factor (*SF*) is the carcinogenic slope factor of the carcinogenic pollutant (mg·kg^−1^·day^−1^). *SF* of Cd, As, Cr and Pb are 6.10, 1.50, 0.50 and 0.0085 mg·kg^−1^·day^−1^, respectively^38^. *TCR* is the sum of the cancer risk index (*CR*) of the individual toxic trace elements. As mentioned above, *TCR* is the sum of *CR*s of Cd, As, Cr and Pb, as they are carcinogenic elements.

5 $$ CR_{i} = ADD_{i} \times SF_{i} $$

6 $$ TCR = \mathop \sum \limits_{j = 1}^{4} CR_{i} $$

### Statistical analysis

The statistical characteristics of the trace element contents of soils and crops in three farmlands were expressed as count distribution, including minimum and maximum values, mean, and standard deviation (*SD*). The exceedance rates were also included to show the extent of trace element contamination for both the farmland soils and crops. One-way analysis of variance (ANOVA) was performed to examine the differences in trace element contents, CEC, pH and SOM among three types of farmland soils and crops. If the variance of the above indicators was homogeneous, then least-square mean separation with Duncan’s correction was used to test the differences among different soils or crops. Otherwise, if the variance was heterogeneous, Tamhane’s T3 test was used to test the differences. Principal component analysis (PCA) and Pearson correlation were used to identify the major source of trace element pollution in the three types of farmland soils. Generalized linear models (GLM) were used to test the potential relationship among soil properties, plant properties, and trace elements in soils and crops. Plant properties included crop type (grain, vegetables, melon and fruit), and growth time. Growth time was the length of time from crop planting to maturity, which was obtained from the *Flora of China* database (http://frps.iplant.cn/) and farmer surveys (supporting information Table S1). In all process of data analysis, the limits of detection (*LOD*) values were assigned to the trace element content when concentrations of the given samples were below *LOD*. All statistical analyses were conducted using the R software package (Version 3.4.2).

## Results

### Trace element contamination status and source identification of farmland soils

The statistical characteristics of the trace element contents in the three types of farmland soils are detailed in Table 1. Unlike the seven metals (Cd, Cr, Cu, Hg, Ni, Pb and Zn) (*p* > 0.05), Kolmogorov–Smirnov test showed As was not complied with normal distribution (*p* < 0.05). The standard deviation (SD) in As seem remarkably high (Table 1). These are probably due to the wide range of locations for the samples collected. Difference in the soil trace element contents derived from rock in soil among different places might cause high *SD* and non-normal distribution. The contents of trace elements differed among the three farmland soils. Specifically, the mean soil contents of As, Cd, Cr, Hg, Pb and Zn in the grain fields were larger than their background values, the exceedance rate for these elements were 46.00%, 80.95%, 79.36%, 80.95%, 90.47% and 88.89%, respectively. For orchard soils, only 4 of trace element contents were higher than soil background values (for As, Cd, Cr and Pb at 11.21, 0.15, 43.01 and 18.83 mg·kg^−1^ respectively). The exceedance rates of these elements were 40%, 92.6%, 72.6% and 100%, respectively (Table 1). In vegetable patch soils, the mean contents of Cd, Cr, Hg, Pb and Zn exceeded soil background values and their exceedance rates were 82.49%, 55.49%, 96.73%, 93.77% and 81.90%, respectively.

**Table 1:** Statistical analysis of eight trace elements (mg·kg^−1^) and soil properties (pH; CEC, cmol(+)·kg^−1^; and SOM, g·kg^−1^) in three farmland soils. The different capital letters after the Mean values indicate a significant difference (*p* < 0.05) in trace element content or soil properties among the three farmlands using One-way ANOVA, whereas same capital letters show no significant differences (*p* > 0.05). Soil properties had no background values thus “/” are used in the table below.

Farmlands	Trace elements and soil properties	Min	Max	Mean	Background value	Standard deviation (SD)	Exceedance rate (%)
Grain field N = 63	As	0.37	75.3	18.22A	11.20	20.51	46.00
	Cd	0.06	0.36	0.18A	0.12	0.08	80.95
	Cr	20.90	61.90	43.95A	39.60	7.64	79.36
	Cu	13.10	36.20	27.56B	35.80	5.09	1.58
	Hg	0.007	0.18	0.03B	0.02	0.02	80.95
	Ni	12.90	34.20	23.35A	26.40	3.99	12.70
	Pb	2.52	37.50	21.11A	13.50	6.40	90.47
	Zn	46.10	156.00	106.61A	76.80	22.73	88.89
	pH	7.61	14.20	8.31AB	/	3.17	/
	CEC	1.10	24.30	8.29B	/	4.72	/
	SOM	9.04	58.40	21.44B	/	9.13	/
Orchard N = 135	As	3.57	24.10	11.21B	11.20	4.23	40.00
	Cd	0.04	0.24	0.15B	0.12	0.03	92.60
	Cr	34.50	56.40	43.01A	39.60	5.17	72.59
	Cu	13.10	46.10	23.95C	35.80	6.69	6.67
	Hg	0.01	0.05	0.018B	0.02	0.008	62.96
	Ni	16.90	33.40	22.58A	26.40	3.33	14.81
	Pb	14.60	25.50	18.83B	13.50	2.42	100
	Zn	46.10	128.00	75.48B	76.80	22.32	38.52
	pH	7.64	13.40	8.70A	/	1.03	/
	CEC	0.40	14.60	7.17B	/	3.45	/
	SOM	9.41	43.30	23.87B	/	8.56	/
Vegetable patch n = 337	As	0.37	98.70	7.15C	11.20	9.67	13.05
	Cd	0.04	0.32	0.17A	0.12	0.05	82.49
	Cr	20.90	64.30	40.48B	39.60	8.69	55.49
	Cu	20.50	76.00	32.74A	35.80	8.17	26.11
	Hg	0.007	0.25	0.08A	0.02	0.05	96.73
	Ni	12.90	44.47	23.38A	26.40	5.20	29.37
	Pb	2.52	38.80	18.06C	13.50	3.93	93.77
	Zn	59.30	244.00	99.04A	76.80	31.41	81.90
	pH	6.80	14.30	8.91B	/	3.22	/
	CEC	0.40	29.30	15.12A	/	5.30	/
	SOM	3.68	57.45	40.94A	/	29.20	/

**Figure 1:**
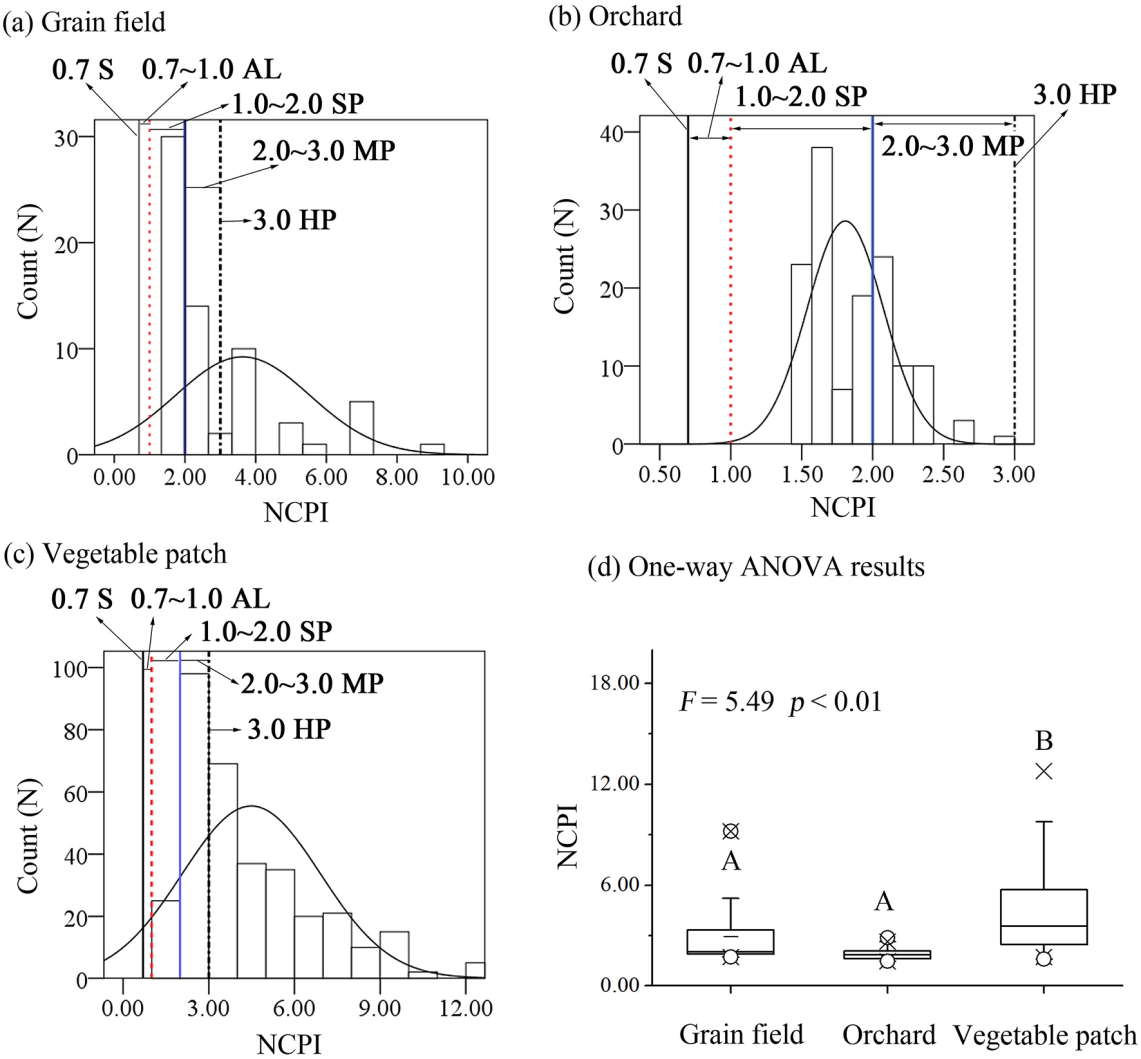
Count distribution and the difference of soil NCPI among three farmland soils. S, AL, SP, MP and HP indicate security, alert level, slight pollution, moderate pollution and heavy pollution, respectively.

The *NCPI* results showed that no soil samples were in the categories of security and warning pollution status (*NCPI* ≤ 1.00), while proportions of soil samples in the slight (1.0 < *NCPI* ≤ 2.0), moderate (2.0 < *NCPI* ≤ 3.0) and heavy (*NCPI* > 3.0) pollution categories accounted for 44.44%, 22.22% and 33.34% of the total soil samples in grain field soils, 64.44%, 35.56% and 0.00% in orchard soils, and 7.42%, 29.08% and 63.50% in vegetable patch soils, respectively (Fig. 1). One-Way ANOVA results showed that Cu, Hg and Zn contents were the highest in vegetable patch soils, whereas As, Cr and Pb contents were highest in grain field soils, but Ni was at similar levels among three farmland soils (Table 1 and supporting information Table S5). The mean value of *NCPI* in vegetable patch soils (4.38 ± 2.42) was significantly higher than that in grain field soils (2.90 ± 1.71) and orchard soils (1.87 ± 0.29) (*p* < 0.05) (Fig. 1).

Pearson correlation and principal component analysis (PCA) results indicated that pollution source of trace elements differed among three farmlands (Table 2). More specifically, in grain field soils, PC1 accounted for 31.88% of the variance and had higher loadings of As, Cd, Pb and Cu. PC2 explained 26.89% of variance and was dominated by Cr and Ni. Hg and Zn composed the greatest loading for PC3, which explained 20.00% of variance. The mean contents of Cu and Ni were lower than the background values (Table 1). After removing the results with content values below the background value, our results found that As, Cd and Pb have the same pollution sources, while Hg and Zn originated from the same sources, but Cr was imported from another source (Tables 1 and 2). In orchard soils, PC1 had higher loadings of Cr, Pb, Cu and Ni, accounting for 45.13% of variance (Tables 1 and 2), whereas PC2 and PC3 contained Cd, Hg, Zn (PC2) and As (PC3), and accounted for 17.00% and 13.32% of variance, respectively. After removing the trace elements with content values below the background value (Cu and Ni in PC1, and As in PC3)(Table 1), Cr and Pb originated from the same source, whereas Cd, Hg and Zn were from another source (Tables 1 and 2). In vegetable patch soils, PC1, PC2 and PC3 accounted for 36.58%, 23.34% and 13.10% of variance, respectively. PC1 had higher loadings of Cd, Cr and Ni, whereas PC2 contained As, Hg and Pb, but PC3 was dominated by Cu and Zn. Due to the low content compared with background values, Cu, Zn and Ni were removed from the principal components (Table 1). Cd and Cr were considered to be from the same source, whereas As, Hg and Pb were produced by a different pollution source (Tables 1 and 2). Within all of the principal components (PC) of three farmland soils, the significance of correlation between all related trace elements was less than 0.05 (Table 2), indicating our classification of the source identification was reasonable.

**Table 2:** Pearson correlation among trace elements and matrix of the principal component analysis (PCA) loadings for three farmland soils. KMO indexes of grain field, orchard, and vegetable patch were 0.68, 0.61 and 0.66 in PCA, respectively.

	Correlation analysis								PCA		
	As	Cd	Cr	Hg	Pb	Cu	Zn	Ni	PC1	PC2	PC3
Grain field									31.88%	26.89%	20.00%
As	1								**0.73**	− 0.38	− 0.46
Cd	0.59**	1							**0.89**	0.08	− 0.14
Cr	− 0.32*	0.02	1						− 0.02	**0.97**	− 0.06
Hg	− 0.19	− 0.13	− 0.31*	1					0.02	− 0.36	**0.78**
Pb	0.74**	0.43**	0.02	− 0.28*	1				**0.68**	− 0.09	− 0.42
Cu	0.40**	0.69**	0.05	0.15	0.09	1			**0.85**	0.14	0.36
Zn	− 0.37**	− 0.21	0.18	0.39**	− 0.22	0.20	1		− 0.01	0.22	**0.75**
Ni	− 0.36**	0.10	0.93*	− 0.20	− 0.13	0.21	0.21	1	0.05	**0.97**	0.10
Orchard									45.13%	17.00%	13.32%
As	1								0.16	0.08	**0.92**
Cd	0.06	1							0.21	**0.75**	0.04
Cr	0.18*	0.31**	1						**0.90**	0.05	0.16
Hg	0.21*	0.52**	0.15	1					− 0.04	**0.85**	0.27
Pb	0.25*	0.30**	0.54**	0.01	1				**0.78**	0.15	0.05
Cu	− 0.04	0.33**	0.41**	0.30**	0.49**	1			**0.60**	0.50	− 0.03
Zn	0.04	0.53**	0.21*	0.49**	0.50**	0.67**	1		0.37	**0.78**	− 0.19
Ni	0.14	0.36**	0.91**	0.30**	0.51**	0.54**	0.45**	1	**0.87**	0.26	0.08
Vegetable patch									35.58%	23.34%	13.10%
As	1								− 0.05	**0.76**	0.18
Cd	0.17**	1							**0.58**	0.40	0.17
Cr	0.02	0.43**	1						**0.94**	− 0.01	− 0.06
Hg	0.20**	0.29**	0.01	1					− 0.04	**0.63**	0.23
Pb	0.49**	0.46**	0.46**	0.29**	1				0.42	**0.73**	− 0.04
Cu	0.20**	0.29**	0.18**	0.39**	0.28**	1			0.16	0.22	**0.87**
Zn	0.42**	0.19**	0.06	0.16**	0.09	0.69**	1		− 0.01	0.16	**0.91**
Ni	− 0.04	0.41**	0.85**	− 0.06	0.40**	0.11*	0.02	1	**0.94**	− 0.09	0.02

All *p* values from Bartlett's Test of Sphericity < 0.001. The eigenvalues > 1 were extracted.

**Correlation is significant at the 0.01 level (2-tailed).

* Correlation is significant at the 0.05 level (2-tailed).

### Trace element contamination status and health risk of crops

About 92% of crop samples contained trace element contents below the Risk Control Standard for China. Trace element contents also differed among grain, vegetables, melon and fruits. According to the Risk Control Standard of trace elements in crops in China issued by the China Health Ministry (Table 3), none of As, Cd, Hg, Cu, Ni, Zn and Pb contents in grains exceeded the standard, whereas 4.76% of the total number of samples with Cr exceeded the standard. In vegetables, the contents of As, Cd, Cu, Hg, Ni and Zn of all samples were lower than the China’s Risk Control Standard, while 1.18% and 0.89% of the samples with Cr and Pb were exceeded, respectively.

**Table 3:** Statistical analysis of eight trace elements in three crops. The different capital letters after the Mean values indicate a significant difference (*p* < 0.05) in trace element content or soil properties among the three farmlands using One-way ANOVA, whereas same capital letters show no significant differences (*p* > 0.05). Risk Control Standard (RCS) is regulated by the China Health Ministry (supporting information Table S7).

Farmlands	Trace elements	Min (mg kg^−1^)	Max (mg kg^−1^)	Mean (mg kg^−1^)	RCS	SD (mg kg^−1^)	Exceedance rate (%)
Grain N = 63	As	< 0.01	0.08	1.78 × 10^–2^ A	≤ 0.70	1.48 × 10^–2^	0.00
	Cd	< 0.001	0.02	3.17 × 10^–3^ A	≤ 0.05	4.42 × 10^–3^	0.00
	Cr	0.03	1.98	0.37 B	≤ 1.00	0.34	4.76
	Cu	0.52	2.39	1.48 A	≤ 10.00	0.32	0.00
	Hg	< 0.003	0.01	2.13 × 10^–3^ A	≤ 0.02	2.92 × 10^–3^	0.00
	Ni	< 0.05	0.05	0.03 × 10^–2^ B	≤ 0.40	0.01	0.00
	Pb	< 0.02	0.20	4.01 × 10^–2^ B	≤ 0.40	4.81 × 10^–2^	0.00
	Zn	0.73	15.80	10.21 A	≤ 50.00	4.10	0.00
Melon and fruits N = 135	As	< 0.01	0.15	1.68 × 10^–2^ A	≤ 0.50	1.94 × 10^–2^	0.00
	Cd	< 0.001	0.07	4.03 × 10^–3^ A	≤ 0.03	8.67 × 10^–3^	2.22
	Cr	< 0.05	3.44	0.26 B	≤ 0.50	0.34	11.11
	Cu	< 0.20	3.47	1.06 A	≤ 10.00	0.53	0.00
	Hg	< 0.003	0.01	1.50 × 10^–3^ A	≤ 0.01	2.23 × 10^–3^	0.00
	Ni	< 0.05	0.33	0.02 A	≤ 0.40	0.06	0.00
	Pb	< 0.02	0.18	0.06 AB	≤ 0.20	0.04	0.00
	Zn	0.23	6.12	1.36 C	≤ 5.00	1.08	1.48
Vegetables n = 337	As	< 0.01	0.31	0.02 A	≤ 0.50	0.03	0.00
	Cd	< 0.001	0.04	8.33 × 10^–3^ B	≤ 0.05	7.56 × 10^–3^	0.00
	Cr	< 0.20	0.73	0.09 A	≤ 0.50	0.08	1.18
	Cu	< 0.003	3.22	0.94 A	≤ 10.00	0.56	0.00
	Hg	< 0.05	0.64*10–2	1.76 × 10^–4^ B	≤ 0.01	0.85 × 10^–4^	0.00
	Ni	< 0.02	0.15	2.84 × 10^–2^ B	≤ 0.40	1.71 × 10^–2^	0.00
	Pb	< 0.02	0.26	0.06 A	≤ 0.20	0.04	0.89
	Zn	0.49	8.37	2.99 B	≤ 20.00	1.31	0.00

One-Way ANOVA results showed that Cd and Hg contents were significantly higher in grain, followed by the contents in melon and fruits, with the lowest in vegetables. In contrast, Cr and Pb contents were the highest in vegetables, followed by the contents in melon and fruits, and the lowest in grain. Zn was highest in grain, followed by vegetables, and the lowest in melon and fruits. As and Cu content were not significantly different among three crops (*p* < 0.05) (Table 3 and supporting information Table S5).

**Figure 2:**
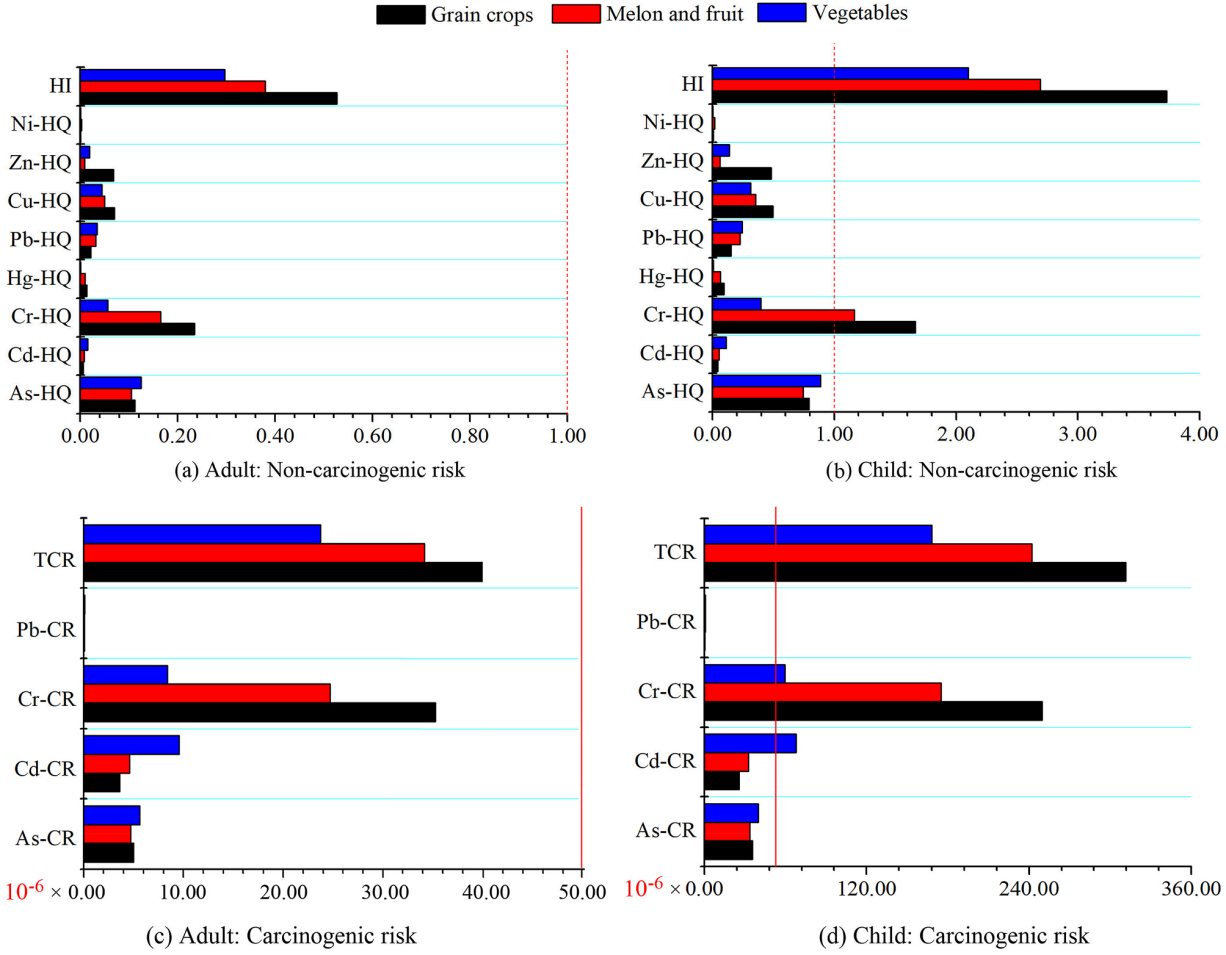
Non-carcinogenic and carcinogenic risks of three types of crops for adults and children. Red lines are thresholds of occurrence of potential non- and carcinogenic risks. The thresholds of non-carcinogenic and carcinogenic risks are 1.0 and 5.0 × 10^–5^.

There was no non-carcinogenic risk for adults based on the *HI*s and *HQ*s in the various trace elements of three crops, *i.e.*, all *HQ*s were < 1 and *HIs* were < 1 (Fig. 2). However, for children, the *HI*s of the three crops were > 1 (Fig. 2). Among the eight trace elements, Cr has the largest *HQ*, additionally its *HQ*s were > 1 for grain, melon and fruits for children (Fig. 2).

Among three crops, grain has the highest *HI*s for both adults and children, whereas melon and fruits were moderate, and vegetables were the lowest (Fig. 2). *CR*s of Pb, Cr, Cd and As in three types of crops for adults, were all below the maximum acceptable risk level of the International Commission of Radiological Protection (5.00 × 10^–5^) (Fig. 2). *TCRs* of three types of crops in adults was also < 5.00 × 10^–5^ (Fig. 2). However, *TCRs* of three crops for children were > 5.00 × 10^–5^. Among four carcinogenic elements, the carcinogenic risks of Cr were obviously higher than those of Cd, As and Pb for three crops (Fig. 2). *CR*s of Cr in three crops were > 5.00 × 10^–5^ for children (Fig. 2). *CR* of Cd in vegetables was > 5.00 × 10^–5^ for children, while grain, melon and fruits were not (Fig. 2). *CR* of Cr in grain for children was higher than that in vegetables, melon and fruits (Fig. 2).

### Influencing factors of trace element accumulation in crops

**Figure 3:**
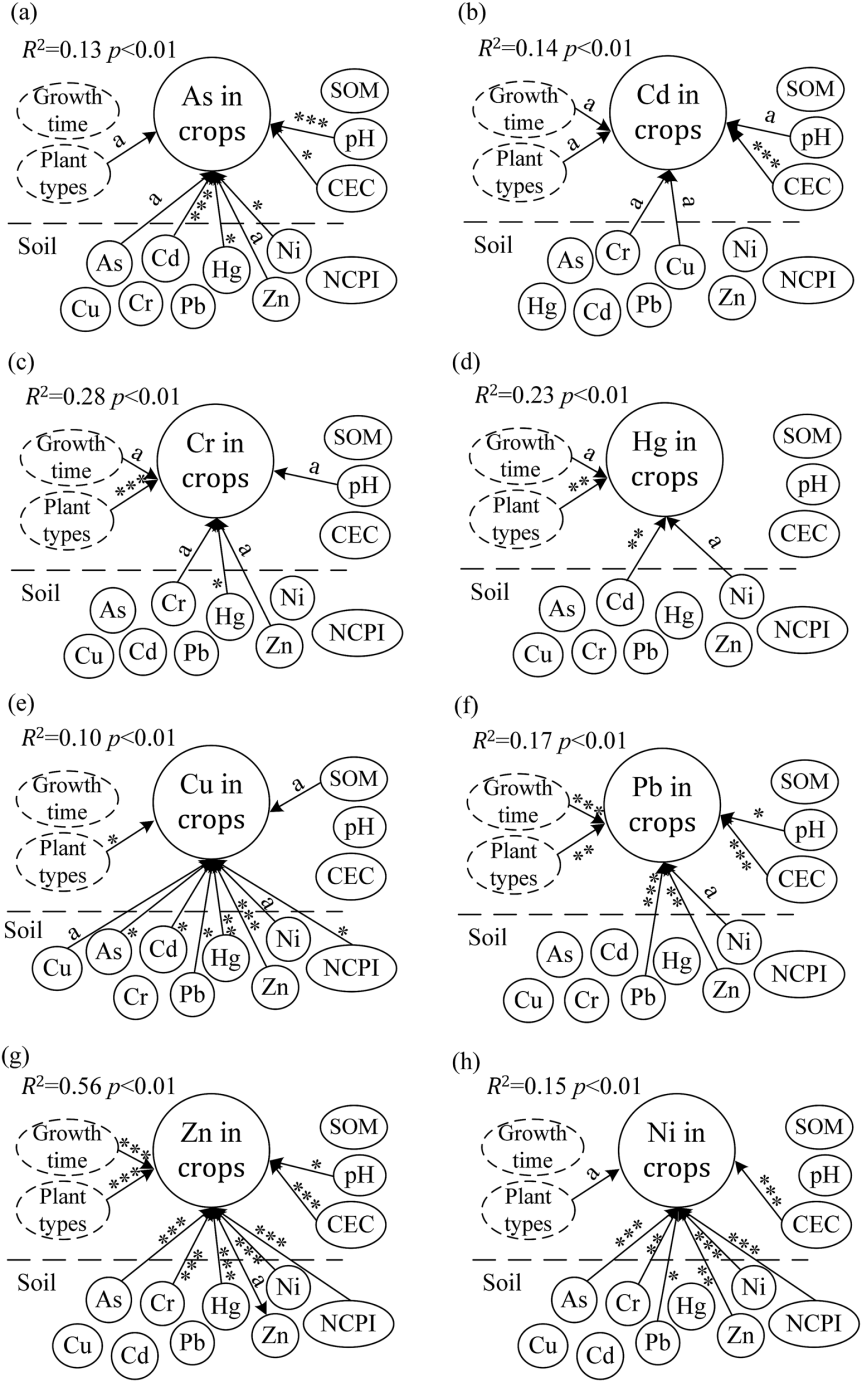
The relationships of soil trace elements, soil physicochemical properties, plant types and growth time against trace elements in crop calculated using generalized linear models (GLM). Arrows indicate that the factors have significant effects on trace elements in crops. ***p < 0.001; **p < 0.01; *p < 0.05;^a^*p* < 0.1.

GLM results showed that all *R*^2^ of regression equation > 0.10, while all *p* value < 0.001. The content of As, Cr, Cu, Pb, Zn and Ni in the crops were positively correlated with their content in farmland soils (*p* < 0.1), whereas Cd and Hg contents correlations were not significant (*p* > 0.1). The number of the significant correlated pairs in relationships of pH, CEC, SOM and soil NCPI against crop trace element content were 5, 5, 1 and 3, respectively (*p* < 0.1). Additionally, plant type was found significantly related with eight trace element contents in crops (*p* < 0.1). Whereas growth time was also significantly related with the contents of Cd, Cr, Hg, Pb, and Zn in crops (*p* < 0.1) (Fig. 3 and Supporting information Table S8).

## Discussion

### Differences in trace element contamination status and pollution risk among three farmland soils

*NCPI* results of all three farmland soils were higher than 1 (Fig. 1), suggesting all soil samples suffered from trace element contamination. This was consistent with previous studies that remarkable industrialisation and urbanisation, and the overuse of pesticides and fertilisers, caused soil trace element pollution in Xinjiang^1,39,40^. The exceedance rates of Cd, Cr, Hg, Pb and Zn were higher than that of As, Cu, and Ni in three farmland soils (Table 1), suggesting they were the major pollutants. This was due to the fact that most of these elements in farmland were introduced from anthropogenic activities and easily affected by human^1,2,13^. For example, automobile exhaust and industrial emissions, waste incineration, sewage irrigation and the overuse of fertilizers and pesticides all increased their contents in farmland^1,10,13,39^.

Cr, Cu, Hg, Pb, Zn and Ni contents in all farmland soils were below the Risk Control Standard of soil trace element contamination of agricultural land in China (GB 15618-2018) (details in supporting information Table S6)^5^, whereas a few samples were exceeded for As and Cd (< 5%). These showed that although most of soils were contaminated by trace elements, the pollution status does not reach harmful levels to human health. In fact, compared with other industrial developed areas in China, trace element contents in farmland soils of Xinjiang was low^1,7,21,29^. For example, Zn, Pb, Cr and Cd contents in farmland soils of Beijing were 81.8, 18.5, 75.7 and 0.18 mg·kg^−1^, respectively^6^. The Cu, Zn, Pb, Cr, Hg and Cd contents in farmland soils of Shanghai were 31.4, 106.2, 26.4, 85.6, 0.13 and 0.20 mg·kg^−1^, respectively^6^. The contents of Cu, Zn, Pb and Cd in farmland soils of Nanchang were 33.2, 87.6, 45.5 and 0.66 mg·kg^−1^, respectively^6^. This difference may be attributed to the lower levels of economic development and shorter cultivation periods^2,3^. Xinjiang is located in an economically underdeveloped region in the inland northwest, China, the lower industrial and traffic conditions introduced less trace elements into the farmland soils compared with the central and eastern China^3,41^. In addition, most of cultivation periods in Xinjiang farmland were shorter than 30 years as major land development along with population migration was carried out after 1980s^29,42^. The shorter cultivation periods also resulted in fewer trace elements into farmland soils.

Our results showed that NCPI varied significantly among three farmland soils. Vegetable patch soil has higher pollution level compared with the other farmland soils (Fig. 1). This may be caused by the differences in planting modes among the three farmlands^3,9,43^. In order to increase yields and control pests, farmers have applied more manure fertilisers and pesticides, resulting in a higher level of soil trace element pollution in vegetable patch^40,43,44^. One-way ANOVA results showed that the contents of seven trace elements were significantly different among three farmland soils except Ni (Table 1 and supporting information Table S5). This may be due to the multifactor functioning such as pollution source and the interaction between plants and soil^1,3,20^. For example, Zhu et al.^45^, has shown that variance in plant accumulation capacity may cause variability of soil trace element content among different farmlands. Different pollution sources can also cause changes in trace element content of farmland soils between different villages^1^. In this study, the PCA and Pearson correlation results indicated different pollution sources of trace elements among farmlands (Table 2). Use of plastic mulch and chemical fertiliser may have resulted in an increase of Cd, As and Pb in grain fields as these metals may be introduced during production of the plastics and fertilisers^46,47^. The intensive agricultural mechanisation may also have contributed to soil Hg and Zn accumulation^14,48^. The grain was mainly cultivated using mechanisation given the larger areas of farmland in Xinjiang. Large diesel- or gasoline-powered dispensers were used to harvest crops and spray pesticides. In these process, Hg and Zn from farm machinery tyres were likely added to soil^1,20^. Cr may have accumulated through the atmospheric settlement and irrigation^14,48^. In orchards, Cr and Pb concentrations were strongly correlated indicating same source (Table 2). It is recognised that Cr and Pb may have originated from atmospheric settlement and transportation^1,9,14^. The orchards are typically closer to human settlements compared with grain fields and vegetable patches, leading to that they are often close to industrial waste gases and automobile exhaust. Similar to grain soils, Cd, Hg and Zn in orchards were mainly introduced from the mechanised planting in large areas^18,44^. The use of large diesel- or gasoline-powered dispensers to spray pesticides and apply phosphate fertiliser also contributed to the input of Cd into soils^1,45,48^. Whereas, in vegetable patches, high trace element content was likely due to the overuse of manure and pesticides, as vegetables were vulnerable to pests and diseases. For example, organic fertilisers such as livestock and poultry feces were found to contain large amounts of Cd and Cr^24,49^. As, Hg and Pb were common constituents of many pesticides, such as Chlorothalonil and Tuzet^1,14^.

### Differences in trace element contamination status and health risks among three crops

The contents of As, Cu, Hg, Zn and Ni of all crop samples were lower than the Risk Control Standard in China, whereas a few samples of Cd, Cr and Pb exceeded the standard levels (Table 3). Similar result was reported from some previous studies that the vast majority of crop samples in Xinjiang were safe for human consumption^1,40,50^. However, trace element contents and health risks varied among the three types of crops (Table 3 and Fig. 2). These differences may be attributed to different crop environmental adaptability and their capacity of element-enrichment^20,21,51^. For example, grain is known for having larger enrichment coefficient for Cr, Hg and Zn, whereas vegetables having a higher capacity for enrichment for Cd and Pb (Supporting information Fig. S2). Besides, varied agricultural activities, soil physiochemical properties and soil trace element contents also probably have also contributed to the variability in crop trace element contents and health risks^11,14,26,43^. In fact, we found higher content of Cd in grain field resulted in more Cd absorbed into grains than vegetables, melon and fruits (Fig. 1 and Table 3).

*HI*s and *HQ*s results showed that children were more susceptive for potential non-carcinogenic risks than adults. At the early stage of life, the functions of various tissues and organs, especially the detoxification and excretion functions of the liver and kidney, were relatively weaker because they are still in the period of growth and development. Thus, children are more sensitive to the toxic effects of trace elements^12^. Among eight trace elements, Cr has the largest *HQ*, and its *HQ*s were > 1 for three crops for children, suggesting it was the major contributor to the non-carcinogenic risk for children (Fig. 2). The high *HQ* of Cr might cause abnormal thyroid artery, polycythaemia, over production of red blood cells and right coronary artery problems for children after the ingestion of crops^52^.

The *CR*s results indicated that the edible part of crops in Xinjiang will not cause a cancer risk to adults, while crop ingestion may cause unacceptable risks for children^1^. Additionally, Cr has the largest carcinogenic risks compared with the other carcinogenic metals. These results suggest that Cr was the major contributor to carcinogenic risks for children^1,9^. The ingestion of local crops might cause lung cancer and nasopharyngeal cancers for children due to high *CR* of Cr^52^. The major cause of Cr carcinogenic risks (CRs of Cr in three types of crops were > 5.00 × 10^–5^) may be related to the overuse of pesticides and mixed farmyard manure^8,9,37^. *HI*s, *TCR*s and *CR* of Cr in grains were much higher than other crops (Fig. 2). These indicated that ingestions of grains had higher carcinogenic risk than vegetables, melon and fruits (Fig. 2). This might be explained by the difference of physiological properties among three crops. As grain, in this case corn, has a longer growth time, allowing more Cr from soil be accumulated in it (supporting information Table S1)^18,44^. Corn also has a higher dry matter ratio compared with vegetables, melon and fruits, hence they were able to accumulate more Cr within their body due to higher conception of grain on dry weight^37,44^. This result contradict with previous finding that vegetables, especially leafy vegetable, post a higher health risk than grain because of faster growth rate and larger absorbing ability of trace elements^43,44,53^. This is probably due to the different influence of soil environment on plant properties between our studied and the other regions. For example, in the low pH soil environment, which is the typical object of most of previous studies, the abundant hydrogen ions would promote the release of trace element ions from hydroxyl surface of clay minerals, thus increasing the quantity and activity of trace element ions^22,23^. The advantages of growth rate and absorbing ability guarantee vegetables to obtain more trace element ions from soil, make a higher health risk in it compared with grain^53^. On the contrary, in the Xinjiang soil containing high saline-alkali (pH ≥ 6.8 in this study), trace element ions will be steadily adsorbed on the surface of clay minerals due to high soil pH, very difficult for plant to intake, constrain the ability to accumulate them in the fast growing vegetables^23^. However, a longer growth time would be beneficial for grain accumulating more trace elements in their body^37,44^. In order to reduce the carcinogenic and non-carcinogenic risks, we recommend diet management of children in Xinjiang should be implemented. Consumption of vegetables, fruits and melon may be increased, while intakes of grains should be reduced.

### Investigation of influencing factors for trace element accumulation in crops

GLM results showed that the contents of As, Cr, Cu, Pb, Zn and Ni in farmland soils determined their contents in crops, while soil Cd and Hg did not (Fig. 3 and Supporting information Table S8). The soil Cd and Hg concentration is not the determining factors for crop concentration is due to the low needs and storing capacity in plant body^18,19,43^. In this study, Cd and Hg were the elements with the lowest concentrations in crops (Table 3), hence, their limited absorption from farmland soil may have little impact on crops. This can also be evidenced by the number of pairs of trace elements that significantly correlated both in soil and crops. The crop Cr and Hg contents have the minimum number of trace element pairs that significantly correlated in both soil and crops (both numbers were 2, whereas for crop As, Cr, Cu, Pb, Zn and Ni contents, the pair numbers were 5, 3, 7, 3, 5 and 5, respectively) (*p* < 0.1) (Fig. 3 and Supporting information Table S8). This result indicated that crop Cr and Hg were not easily affected by other soil metallic elements potentially due to their low contents. In addition, connection between the content of a given trace element in crops and other soil element contents varied with the types of trace elements. For example, the As content in crops was significantly correlated with soil Cd, Hg, Zn and Ni (*p* < 0.1), but was not associated with soil Cr, Cu and Pb. The Cd content in crops was significantly associated with soil Cr, Hg and Cu (*p* < 0.1), whereas it was not correlated with the rest of the soil trace elements (*p* > 0.1) (Fig. 3 and Supporting information Table S8). This indicated that complex correlations existed in trace elements between soil and crops, which may be caused by competition between elements or plant selectivity for trace elements, and pollution source of soil trace elements^13,15,19^. A given trace element in crops may have a similar relationship with these elements where it has originated from the same pollution source^48^.

Trace element contents in the edible part of crops were also affected by pH, SOM, CEC, and soil contamination status (NCPI) (Fig. 3 and Supporting information Table S8). This was most probably because pH, SOM, CEC and NCPI affect the valence of soil trace elements and the absorption capacity of plant roots^26,27^. CEC and pH were significantly correlated with five trace elements in crops (pH: As, Cd, Cr, Pb and Zn; CEC: As, Cd, Pb, Zn and Ni), while NCPI and SOM were associated with three (Cu, Zn and Ni) and one (Cu) metals, respectively. These indicated that the influence of pH and CEC on trace element content in crops is higher than that of SOM and NCPI. Among these biotic and abiotic properties, plant type was found significantly related with all trace element contents in crops (*p* < 0.1), indicating that, compared with soil trace element contents and soil properties, it might be more effective in reducing health risks caused by trace element pollution. This finding is novelty and was rarely considered in the practice for controlling trace element contamination in crops^2,11,54^. Currently, large number of studies consider that soil trace element pollution is the most negative important factor of crop safety^4,6,8,54^. Reducing trace element contents in farmland was often seen as the primary method for minimizing the health risks associated with consumption of metal and metalloid contaminated crops^3,6,54^. In this study, we identified that plant type has a greater effect on the trace element contents in the edible part of the crop than soil trace element contents (Fig. 3 and Supporting information Table S8). Which suggested that, planting different crops in trace element contaminated soils might be an effective approach to reduce the health risks. For example, in saline-alkali soils of our studied regions, cultivating vegetables and fruits on high trace element contaminated soils, while planting grains on low trace element polluted soil might may decrease potential risk to human health via food intake. However, *R*^2^ of our results of GLM were relatively low, except for Zn. This may also indicate that the content of crop trace elements was determined by the combined action of multiple factors^25,27,33^. More work needs to be done in the future to clarify trace element contents in crops and their influencing factors.

## Conclusions

The 535 sampling points were selected to study whether trace element contamination status and health risks differed among three farmlands and three crops, and which factors affecting the change of trace element contents among crop in Xinjiang. The results showed that majority of crop samples (91.97%) were safe in terms of trace element contents based on China’s Risk Control Standard, whereas all farmland soil samples were considered to be polluted. Grains from sampling sites represented higher non-carcinogenic and carcinogenic risks compared with vegetable, melon and fruits, but pollution levels in their grown soils showed the opposite pattern. The ingestion of grains brought higher non-carcinogenic and carcinogenic risks than vegetables, melons and fruits. Non-carcinogenic and carcinogenic risks associated with various trace elements caused by the ingestion of the edible part of crops for children were higher than adults. Increasing the consumption of vegetables, fruits and grains, while reducing the intakes of grains, should be adopted to reduce the carcinogenic and non-carcinogenic risks for children. The contents of As, Cr, Cu, Pb, Zn and Ni in the edible part of crops were found to be affected by its corresponding soil content, while Cd and Hg were not. pH, SOM, CEC, soil NCPI, plant type and growth time also have influences on the trace element content in the edible part of crop. Compared with other factors, plant type was more effective in reducing health risks caused by trace element pollution, indicating that planting different crops in trace element contaminated soils could be an effective way to reduce the health risks. However, Xinjiang is a very big region in China, results obtained from this study (only 535 sites) may not be completely representative of the trace element contamination status for both soils and crops. However, it would be interesting to whether the recommended approach will work or not in practice.

## Supplementary Information

Supplementary Information

## References

[1] Rukeya S (2018). Pollution characteristics and health risk assessment of heavy metals in the vegetable bases of northwest China. Sci. Total Environ..

[2] Li X (2019). Technical solutions for the safe utilization of heavy metal contaminated farmland in China: a critical review. Land Degrad. Dev..

[3] Yang Q (2018). A review of soil heavy metal pollution from industrial and agricultural regions in China: pollution and risk assessment. Sci. Total Environ..

[4] Zhang X, Zhong T, Liu L, Yang X (2015). Impact of Soil heavy metal pollution on food safety in China. PLoS ONE.

[5] Chinese Ministry of Environmental Protection (CMEP). National survey bulletin of soil pollution. Beijing. http://www.gov.cn/foot/2014‐04/17/content_2661768.htm. (2014).

[6] Li J, Wang H (2014). Soil contamination with heavy metals and its impact on food security in China. J. Geo Environ. Prot..

[7] Khan S, Cao Q, Zheng YM, Huang YZ, Zhu YG (2008). Health risks of heavy metals in contaminated soils and food crops irrigated with wastewater in Beijing, China. Environ. Pollut..

[8] Liu X (2013). Human health risk assessment of heavy metals in soil-vegetable system: a multi-medium analysis. Sci. Total Environ..

[9] Yu Y-J (2010). Pollution characteristics and ecological risk assessment of heavy metals in farmland soils of a typical basin. Res. Environ. Sci..

[10] Xiao R (2019). Accumulation, ecological-health risks assessment, and source apportionment of heavy metals in paddy soils: A case study in Hanzhong, Shaanxi, China. Environ. Pollut..

[11] Xu X, Wang T, Sun M, Bai Y, Hagist S (2019). Management principles for heavy metal contaminated farmland based on ecological risk—a case study in the pilot area of Hunan province, China. Sci. Total Environ..

[12] Chunhabundit R (2016). Cadmium exposure and potential health risk from foods in contaminated area, Thailand. Toxicol. Res..

[13] Guo X, Sun Q, Zhao Y, Cai H (2019). Identification and characterisation of heavy metals in farmland soil of Hunchun basin. Environ Geol..

[14] dos, Santos, F. S., Sobrinho, M. B. A., Nelson, M. O. C. Heavy Metals Contamination in Soil and Plants by Intensive Use of Pesticides And Fertilizers. 1441–1447 (2002).

[15] Liu G, Yu Y, Hou J, Xue W, Liu Z (2014). An ecological risk assessment of heavy metal pollution of the agricultural ecosystem near a lead-acid battery factory. Ecol. Indicators.

[16] Toth G, Hermann T, Da Silva MR, Montanarella L (2016). Heavy metals in agricultural soils of the European Union with implications for food safety. Environ. Int..

[17] Dahmani-Muller H, Oort FV, Gélie B, Balabane M (2000). Strategies of heavy metal uptake by three plant species growing near a metal smelter. Environ. Pollut..

[18] Altarawneh RM (2019). Levels of selected heavy metals (Pb, Ni, Cd, and Cr) in various widely consumed fruits and vegetables in Jordan. Int. J. Environ. Anal. Chem..

[19] Fang HW, Li WS, Tu SX, Ding YZ, Feng RW (2019). Differences in cadmium absorption by 71 leaf vegetable varieties from different families and genera and their health risk assessment. Ecotoxicol. Environ. Saf..

[20] Tudi M (2019). Difference of trace element exposed routes and their health risks between agriculture and pastoral areas in Bay County Xinjiang, China. Environ. Sci. Pollut. Res..

[21] Chabukdhara M, Munjal A, Nema AK, Gupta SK, Kaushal RK (2015). Heavy metal contamination in vegetables grown around peri-urban and urban-industrial clusters in Ghaziabad, India. Hum. Ecol. Risk Assess Int. J..

[22] Gestel CAMV (2008). Physico-chemical and biological parameters determine metal bioavailability in soils. Sci. Total Environ..

[23] Kim RY (2015). Bioavailability of heavy metals in soils: definitions and practical implementation—a critical review. Environ. Geochem. Health.

[24] Lambert RL, Grant C, Sauvé S (2007). Cadmium and zinc in soil solution extracts following the application of phosphate fertilizers. Sci. Total Environ..

[25] Tchounwou PB, Raich JW, Yedjou CG, Patlolla AK, Sutton DJ, Luch A (2012). Heavy metal toxicity and the environment. Molecular, Clinical and Environmental Toxicology. Experientia Supplementum vol 101.

[26] Zeng F (2011). The influence of pH and organic matter content in paddy soil on heavy metal availability and their uptake by rice plants. Environ. Pollut..

[27] Walker DJ, Clemente R, Bernal MP (2004). Contrasting effects of manure and compost on soil pH, heavy metal availability and growth of *Chenopodium album* L. in a soil contaminated by pyritic mine waste. Chemosphere.

[28] The Government of Xinjiang Uygur Autonomous Region of China (GX). Geography of Xinjiang*.* Website of the Government of Xinjiang Uygur Autonomous Region of China http://www.xinjiang.gov.cn/xinjiang/qhtz/common_list.shtml (2020).

[29] Li X, Yang X, Gong L (2020). Evaluating the influencing factors of urbanization in the Xinjiang Uygur Autonomous Region over the past 27 years based on VIIRS-DNB and DMSP/OLS nightlight imageries. PLoS ONE.

[30] Nelson DW, Sommers LE (1960). A rapid and accurate procedure for estimation of organic carbon in soils. Proc. Indiana Acad. Sci..

[31] Hendershot WH, Duquette M (1986). A simple barium chloride method for determining cation exchange capacity and exchangeable cations1. Soil Sci. Soc. Am. J..

[32] Yang L, Ai J, Huang W, Liao Z (2015). Determination of 8 heavy metals in vegetables by inductively coupled plasma mass spectrometry (in Chinese with English abstract). J. Anhui Agri. Sci..

[33] Yang J, Ma S, Zhou J, Song Y, Li F (2018). Heavy metal contamination in soils and vegetables and health risk assessment of inhabitants in daye, China. J. Int. Med. Res..

[34] National Environmental Monitoring Centre of China (NEMC) (1990). China’s Soil Element Background Values.

[35] USEPA (2002). Supplemental Guidance for Developing Soil Screening Levels for Super Fund Sites.

[36] USEPA (1989). Risk Assessment Guidance for Superfund. Vol I. Human Health Evaluation Manual.

[37] Yang W (2019). Heavy metals and associated health risk of wheat grain in a traditional cultivation area of Baoji, Shaanxi, China. Environ. Monit. Assess..

[38] Park RM (2018). Risk assessment for metalworking fluids and cancer outcomes. Am. J. Ind. Med..

[39] Mamat Z, Yimit H, Ji RZA, Eziz M (2014). Source identification and hazardous risk delineation of heavy metal contamination in Yanqi basin, northwest China. Sci. Total Environ..

[40] Zhang W, Chen Y, Qi Y, Hong C (2017). Seasonal variations of mercury levels and human health risk in vegetables from Arid Oasis (Shihezi city), Xinjiang, Northwest China. Hum. Ecol. Risk Assess. Int. J..

[41] Song W, Chen B, Liu L (2013). Soil heavy metal pollution of cultivated land in China. Res. Soil Water Conserv..

[42] Yang X, Ali A, Xu Y, Jiang L, Lv G (2019). Soil moisture and salinity as main drivers of soil respiration across natural xeromorphic vegetation and agricultural lands in an arid desert region. CATENA.

[43] Lavado RS, Porcelli CA, Alvarez R (2001). Nutrient and heavy metal concentration and distribution in corn, soybean and wheat as affected by different tillage systems in the Argentine Pampas. Soil Till. Res..

[44] Ali MHH, Al-Qahtani KM (2012). Assessment of some heavy metals in vegetables, cereals and fruits in Saudi Arabian markets. Egypt. J. Aquat. Res..

[45] Zhu G (2018). Heavy metal contents and enrichment characteristics of dominant plants in wasteland of the downstream of a lead-zinc mining area in Guangxi, Southwest China. Ecotoxicol. Environ. Saf..

[46] Mortvedt JJ (1995). Heavy metal contaminants in inorganic and organic fertilizers. Fertil. Res..

[47] Taber, H. G. & Ennis, R. Plant uptake of heavy metals from decomposition of plastigone (TM) photodegradable plastic mulch. In Twenty-First National Agricultural Plastics Conferecne, Orlando, FL, March 5–9, 21, 47–52 (1989).

[48] Li FR, Kang LF, Gao XQ, Hua W, Hei WL (2007). Traffic-related heavy metal accumulation in soils and plants in northwest China. Soil Sediment Contam..

[49] Nookabkaew S, Rangkadilok N, Prachoom N, Satayavivad J (2016). Concentrations of trace elements in organic fertilizers and animal manures and feeds and cadmium contamination in herbal tea (*Gynostemma pentaphyllum* Makino). J. Agric. Food Chem..

[50] Ying F, Wang J (2010). Analysis and assessment of heavy metal pollution of vegetables in Kashgar. J. Anhui Agric. Sci..

[51] Zhou H, Yang W, Zhou X (2016). Accumulation of heavy metals in vegetable species planted in contaminated soils and the health risk assessment. Int. J. Environ. Res. Public Health.

[52] Shanker AK, Cervantes C, Loza-Tavera H, Avudainayagam S (2005). Chromium toxicity in plants. Environ. Int..

[53] Ran J, Ning P, Sun X, Liang D (2019). Heavy metal pollution characteristics and potential risks of soil and crops in Gejiu, Yunnan. Environ. Monit. China.

[54] Hui H, Qian J, Philip K (2014). A study of heavy metal pollution in china: current status, pollution-control policies and countermeasures. Sustainability.

