# Supplementary material for: Variability in plant trace element uptake across different crops, soil contamination levels and soil properties in the Xinjiang Uygur Autonomous Region of northwest China
Source: Sci Rep. 2021 Jan 22;11:2064. doi: 10.1038/s41598-021-81764-w (PMC7822888; doi:10.1038/s41598-021-81764-w)

**Variability in plant trace elements uptake across different crops, soil contamination levels and soil properties in the Xinjiang Uygur Autonomous Region of northwest China**

**Weiguo Liu^1^, Xiaodong Yang^1,2*^, Luchun Duan^3,4^, Ravi Naidu^3,4^, Kaihong Yan^3,4^, Yanju Liu^3,4^, Xiyuan Wang^1,5^, Yongchao Gao^6^, Yinguang Chen^1,7*^**

1 Institute of Resources and Environment Science, Xinjiang University, Urumqi 830046, China; 2 Department of Geography & Spatial Information Technology, Ningbo University, Ningbo 315211, China; 3 Global Centre for Environmental Remediation (GCER), the University of Newcastle (UON), Newcastle, NSW 2308, Australia; 4 Cooperative Research Centre for Contamination Assessment and Remediation of the Environment (CRC CARE), the University of Newcastle, Callaghan Campus, NSW 2308, Australia; 5 Key Laboratory of Oasis Ecology, Urumqi 830046, China; 6 Ecology Institute, Qilu University of Technology (Shandong Academy of Sciences), Shandong Provincial Key Laboratory of Applied Microbiology, Jinan 250306, China; 7 State Key Laboratory of Pollution Control and Resource Reuse, School of Environmental Science and Engineering, Tongji University, Shanghai 200092, China

***Corresponding author:** **Xiaodong Yang**. *Department of Geography & Spatial Information Technology, Ningbo University*, *Ningbo* 315211, *China*; **Tel:** +86-574-87605594; **E-mail address:** [xjyangxd@sina.com](mailto:xjyangxd@sina.com).

**Yinguang Chen.** *State Key Laboratory of Pollution Control and Resource Reuse, School of Environmental Science and Engineering, Tongji University, Shanghai* 200092*, China*; **E-mail address:** [yinguangchen@tongji.edu.cn](mailto:yinguangchen@tongji.edu.cn)

**Postal address:** NO.1188 North ring Road, Department of Geography & Spatial Information Technology, Ningbo University, Ningbo 315211, China.

**Supporting information**

**Table S1** Growth time, sample count and name of 31 crops

| Farmland | Name | Growth time (d) | Sample count (n) |
| --- | --- | --- | --- |
| Grain field | Corn | 230 | 63 |
| Orchard | Red date | 120 | 13 |
|  | Pear | 190 | 14 |
|  | Apple | 180 | 13 |
|  | Grape | 185 | 16 |
|  | Muskmelon | 90 | 13 |
|  | Bergamot pear | 180 | 66 |
| Vegetable patch | Chinese cabbage | 100 | 12 |
|  | Cabbage | 80 | 15 |
|  | Green onion | 40 | 15 |
|  | Wax gourd | 120 | 16 |
|  | Long bean | 70 | 11 |
|  | Cherry tomato | 60 | 13 |
|  | Sweet potato | 150 | 21 |
|  | Carrot | 180 | 13 |
|  | Cucumber | 60 | 15 |
|  | Cowpea | 70 | 13 |
|  | Leek | 40 | 12 |
|  | Bitter gourd | 70 | 10 |
|  | Pepper | 60 | 21 |
|  | Pumpkin | 120 | 13 |
|  | Eggplant | 60 | 21 |
|  | Celery | 60 | 9 |
|  | Green turnip | 70 | 15 |
|  | Lettuce | 30 | 16 |
|  | Towel gourd | 75 | 12 |
|  | Sugar beet | 180 | 12 |
|  | Tomato | 60 | 15 |
|  | Coriander | 50 | 10 |
|  | Pakchoi cabbage | 35 | 18 |
|  | Leaf lettuce | 35 | 9 |

**Table S2** Soil trace element contents in three farmlands (mg·kg^-1^). Data are showed as Mean ± SD.

| Farmland | Crop species | As | Cd | Cr | Hg | Pb | Cu | Zn | Ni | NCPI |
| --- | --- | --- | --- | --- | --- | --- | --- | --- | --- | --- |
| Grain field | Corn | 18.23±20.51 | 0.19±0.08 | 43.95±7.63 | 0.03±0.03 | 21.11±6.40 | 27.56±5.09 | 106.62±22.73 | 23.34±3.99 | 2.95±1.73 |
| Orchard | Red date | 12.80±4.99 | 0.14±0.04 | 46.43±7.52 | 0.03±0.01 | 18.17±1.31 | 26.17±8.14 | 77.85±18.73 | 25.57±4.96 | 4.85±2.88 |
|  | Pear | 13.94±0.97 | 0.18±0.02 | 47.68±1.63 | 0.03±0.01 | 18.69±1.90 | 25.85±1.90 | 82.15±4.73 | 26.73±1.52 | 2.06±0.15 |
|  | Apple | 15.24±2.04 | 0.18±0.05 | 42.38±5.36 | 0.03±0.01 | 20.21±3.02 | 25.69±2.53 | 106.59±23.51 | 23.67±3.67 | 2.30±0.31 |
|  | Grape | 9.06±4.01 | 0.16±0.02 | 42.38±0.41 | 0.03±0.01 | 19.64±1.00 | 28.45±2.26 | 122.86±6.96 | 24.92±0.74 | 2.09±0.14 |
|  | Muskmelon | 9.15±5.05 | 0.17±0.02 | 42.37±2.57 | 0.04±0.02 | 18.03±1.50 | 28.83±7.67 | 71.53±7.82 | 22.83±1.15 | 2.28±0.57 |
|  | Bergamot pear | 10.40±4.07 | 0.15±0.02 | 42.28±4.88 | 0.02±0.01 | 18.70±2.55 | 22.70±7.05 | 66.59±14.67 | 21.44±2.55 | 1.75 ±0.23 |
| Vegetable patch | Chinese cabbage | 5.91±3.92 | 0.17±0.05 | 37.87±9.08 | 0.06±0.04 | 16.73±3.32 | 30.54±4.05 | 92.64±21.03 | 21.76±4.93 | 3.62±1.69 |
|  | Cabbage | 4.94±1.77 | 0.17±0.04 | 43.33±5.10 | 0.07±0.03 | 18.53±0.97 | 30.38±4.79 | 89.03±16.46 | 25.80±3.12 | 3.66±1.37 |
|  | Green onion | 5.63±2.86 | 0.17±0.04 | 45.11±8.92 | 0.08±0.06 | 17.69±4.41 | 31.91±6.87 | 100.53±26.75 | 26.15±4.71 | 4.47±3.07 |
|  | Wax gourd | 6.45±0.29 | 0.18±0.02 | 41.40±1.21 | 0.11±0.06 | 20.37±0.58 | 37.77±6.47 | 139.67±16.16 | 23.13±1.96 | 5.93±3.18 |
|  | Long bean | 7.35±5.68 | 0.16±0.08 | 32.24±6.52 | 0.07±0.05 | 16.52±6.85 | 37.70±17.44 | 125.73±59.06 | 19.31±4.52 | 4.30±1.90 |
|  | Cherry tomato | 8.34±2.70 | 0.14±0.04 | 40.73±12.79 | 0.08±0.06 | 13.85±7.69 | 31.32±3.37 | 105.48±31.28 | 24.13±7.02 | 4.19±3.09 |
|  | Sweet potato | 8.90±5.67 | 0.19±0.04 | 39.43±7.20 | 0.12±0.05 | 18.93±2.81 | 34.60±7.19 | 89.57±18.29 | 21.61±3.00 | 6.04±2.64 |
|  | Carrot | 9.20±6.92 | 0.20±0.02 | 46.14±11.87 | 0.09±0.06 | 18.24±3.32 | 32.74±5.78 | 104.06±29.66 | 26.64±6.85 | 4.85±2.88 |
|  | Cucumber | 4.89±1.70 | 0.16±0.04 | 41.27±9.81 | 0.09±0.03 | 18.90±2.23 | 33.77±5.04 | 89.43±12.86 | 2.53±4.04 | 4.86±1.55 |
|  | Cowpea | 5.28±2.82 | 0.16±0.04 | 39.00±7.87 | 0.07±0.05 | 18.36±3.53 | 35.41±9.62 | 97.93±31.74 | 23.08±4.69 | 3.95 ±2.17 |
|  | Leek | 4.77±2.22 | 0.15±0.06 | 50.17±6.99 | 0.06±0.06 | 17.37±3.06 | 30.10±1.65 | 97.37±24.61 | 26.77±1.95 | 3.28±2.57 |
|  | Bitter gourd | 6.41±6.18 | 0.15±0.03 | 33.80±5.83 | 0.08±0.04 | 16.97±2.12 | 31.18±3.39 | 96.56±21.27 | 19.70±3.79 | 4.47±1.92 |
|  | Pepper | 5.73±3.88 | 0.16±0.04 | 41.64±8.30 | 0.08±0.04 | 19.23±4.03 | 31.14±6.78 | 99.51±29.46 | 23.84±4.68 | 4.13±2.16 |
|  | Pumpkin | 8.33±6.67 | 0.18±0.20 | 43.81±5.64 | 0.08±0.06 | 18.83±1.43 | 31.11±4.32 | 100.04±27.90 | 24.64±2.62 | 4.39±2.91 |
|  | Eggplant | 7.32±5.56 | 0.15±0.05 | 35.07±6.78 | 0.09±0.06 | 18.31±8.22 | 31.83±5.70 | 109.36±46.96 | 19.97±3.66 | 4.93 ±2.73 |
|  | Celery | 8.70±4.58 | 0.19±0.03 | 41.48±6.73 | 0.10±0.06 | 18.81±2.40 | 35.36±4.63 | 114.59±26.66 | 23.47±4.01 | 5.40±3.17 |
|  | Green turnip | 5.13±3.48 | 0.15±0.04 | 40.67±8.84 | 0.08±0.06 | 17.26±2.65 | 29.79±6.43 | 90.31±26.22 | 23.50±4.91 | 4.28±2.83 |
|  | Lettuce | 5.69±6.33 | 0.15±0.02 | 28.73±5.42 | 0.08±0.00 | 13.00±1.38 | 42.33±29.15 | 157.33±75.05 | 15.40±1.39 | 4.36±0.32 |
|  | Towel gourd | 4.56±1.97 | 0.15±0.03 | 44.91±11.40 | 0.07±0.05 | 19.35±2.50 | 34.28±7.20 | 93.21±18.72 | 26.07±6.35 | 3.90±2.42 |
|  | Sugar beet | 13.40±3.00 | 0.17±0.00 | 44.50±6.44 | 0.03±0.03 | 17.06±1.46 | 25.30±5.78 | 117.00±30.55 | 44.47±6.67 | 2.13±0.05 |
|  | Tomato | 6.98±5.56 | 0.16±0.07 | 42.01±8.63 | 0.06±0.04 | 18.46±2.44 | 30.31±6.20 | 91.11±20.82 | 23.62±4.36 | 3.34±1.76 |
|  | Coriander | 4.07±2.11 | 0.14±0.04 | 38.42±10.58 | 0.10±0.07 | 15.80±5.14 | 29.89±7.15 | 97.38±33.02 | 22.52±5.93 | 5.56±3.32 |
|  | Pakchoi cabbage | 9.61±3.37 | 0.17±0.04 | 44.71±9.04 | 0.07±0.05 | 17.73±2.79 | 36.31±19.26 | 110.91±65.26 | 25.42±5.41 | 3.85±2.70 |
|  | Leaf lettuce | 5.51±5.41 | 0.17±0.04 | 33.55±8.58 | 0.07±0.01 | 14.95±2.42 | 38.75±24.85 | 128.60±78.79 | 18.75±5.85 | 3.81±0.78 |

**Table S3** Contents of trace elements in the edible part of crops (mg·kg^-1^). NA represents trace element content lower than the limit of detection *(LOD*). Data are showed as Mean ± SD.

| Crop types | Crop species | As | Cd | Cr | Hg | Pb | Cu | Zn | Ni |
| --- | --- | --- | --- | --- | --- | --- | --- | --- | --- |
| Grain | Corn | 0.02±0.02 | (0.03±0.040×10^-1^ | 0.38±0.34 | (0.02±0.03)×10^-1^ | 0.05±0.05 | 1.18±0.33 | 10.18±4.34 | 0.01±0.02 |
| Melon and fruit | Red date | 0.01±0.01 | (0.04±0.06) ×10^-1^ | 0.15±0.09 | NA | 0.03±0.02 | 2.09±0.69 | 3.99±1.00 | 0.14±0.13 |
|  | Pear | 0.01±0.01 | (0.01±0.01) ×10^-1^ | 0.12±0.09 | NA | 0.04±0.02 | 0.93±0.19 | 0.90±0.24 | 0.09±0.03 |
|  | Apple | 0.02±0.02 | (0.10±0.08) ×10^-2^ | 0.10±0.10 | (0.01±0.02)×10^-1^ | 0.03±0.02 | 0.63±0.21 | 0.44±0.19 | 0.08±0.15 |
|  | Grape | 0.03±0.02 | (0.01±0.07) ×10^-2^ | 0.05±0.06 | NA | 0.03±0.02 | 1.20±0.27 | 0.69±0.13 | (0.09±0.24)×10^-1^ |
|  | Muskmelon | 0.02±0.01 | (0.02±0.02) ×10^-1^ | 0.10±0.04 | NA | 0.04±0.01 | 0.48±0.44 | 1.64±1.02 | NA |
|  | Bergamot pear | 0.02±0.02 | (0.05±0.10) ×10^-1^ | 0.30±0.23 | (0.02±0.02)×10^-1^ | 0.07±0.03 | 1.00±0.37 | 1.20±0.61 | NA |
| Vegetable | Chinese cabbage | 0.03±0.02 | (0.13±0.07) ×10^-1^ | 0.14±0.14 | (0.01±0.01)×10^-1^ | 0.08±0.05 | 0.45±0.13 | 3.13±0.67 | (0.02±0.11)×10^-1^ |
|  | Cabbage | 0.01±0.01 | (0.08±0.08) ×10^-1^ | 0.11±0.12 | NA | 0.06±0.03 | 0.28±0.24 | 2.25±0.94 | NA |
|  | Green onion | 0.02±0.03 | (0.08±0.07) ×10^-1^ | 0.08±0.06 | (0.00±0.01)×10^-1^ | 0.06±0.02 | 0.68±0.24 | 3.39±0.88 | NA |
|  | Wax gourd | 0.01±0.02 | (0.04±0.04) ×10^-1^ | 0.09±0.03 | NA | 0.07±0.02 | 0.11±0.11 | 0.73±0.25 | NA |
|  | Long bean | 0.01±0.02 | (0.02±0.03) ×10^-1^ | 0.05±0.15 | NA | 0.05±0.04 | 0.93±0.37 | 3.95±0.79 | NA |
|  | Cherry tomato | 0.01±0.01 | (0.08±0.010×10^-1^ | 0.21±0.35 | NA | 0.04±0.02 | 0.61±0.17 | 1.59±0.27 | NA |
|  | Sweet potato | 0.02±0.02 | (0.06±0.04) ×10^-1^ | 0.09±0.05 | NA | 0.09±0.05 | 1.53±0.52 | 2.10±0.85 | NA |
|  | Carrot | 0.01±0.01 | (0.12±0.07) ×10^-1^ | 0.06±0.02 | NA | 0.05±0.02 | 0.56±0.22 | 2.56±0.52 | NA |
|  | Cucumber | 0.04±0.04 | (0.04±0.03) ×10^-1^ | 0.08±0.04 | NA | 0.06±0.03 | 0.63±0.25 | 2.11±0.72 | NA |
|  | Cowpea | 0.01±0.01 | (0.05±0.06) ×10^-1^ | 0.07±0.04 | NA | 0.07±0.04 | 1.60±0.43 | 5.18±1.21 | (0.03±0.13)×10^-1^ |
|  | Leek | 0.05±0.04 | (0.07±0.03) ×10^-1^ | 0.09±0.03 | NA | 0.07±0.03 | 0.77±0.16 | 2.18±0.58 | NA |
|  | Bitter gourd | 0.02±0.03 | (0.02±0.01) ×10^-1^ | 0.05±0.04 | NA | 0.03±0.03 | 0.92±0.22 | 2.91±0.75 | NA |
|  | Pepper | 0.01±0.01 | (0.08±0.05) ×10^-1^ | 0.06±0.03 | (0.05±0.01)×10^-1^ | 0.06±0.03 | 1.39±0.50 | 2.78±0.77 | NA |
|  | Pumpkin | 0.02±0.04 | (0.09±0.09)×10^-1^ | 0.07±0.02 | NA | 0.07±0.03 | 1.00±0.43 | 2.58±1.08 | NA |
|  | Eggplant | 0.01±0.02 | (0.07±0.05)×10^-1^ | 0.09±0.07 | NA | 0.06±0.03 | 1.04±0.36 | 2.31±0.79 | NA |
|  | Celery | 0.03±0.03 | (0.13±0.06) ×10^-1^ | 0.10±0.05 | (0.01±0.01)×10^-1^ | 0.09±0.06 | 0.70±0.33 | 4.19±1.37 | NA |
|  | Green turnip | 0.03±0.03 | (0.09±0.06) ×10^-1^ | 0.08±0.04 | NA | 0.05±0.03 | 0.34±0.16 | 2.92±0.75 | 0.01±0.03 |
|  | Lettuce | 0.04±0.03 | (0.14±0.17) ×10^-1^ | 0.08±0.03 | (0.01±0.02)×10^-1^ | 0.05±0.02 | 0.47±0.09 | 2.94±0.11 | NA |
|  | Towel gourd | 0.01±0.02 | (0.04±0.07) ×10^-1^ | 0.08±0.05 | (0.00±0.01)×10^-1^ | 0.05±0.03 | 1.20±0.19 | 3.07±0.81 | NA |
|  | Sugar beet | 0.03±0.01 | (0.02±0.01) ×10^-1^ | 0.19±0.07 | NA | 0.03±0.01 | 0.82±0.22 | 2.02±0.81 | 0.13±0.03 |
|  | Tomato | 0.01±0.01 | 0.01±0.01 | 0.09±0.06 | NA | 0.05±0.02 | 0.70±0.25 | 1.83±0.52 | NA |
|  | Coriander | 0.04±0.03 | 0.02±0.01 | 0.13±0.07 | NA | 0.10±0.08 | 1.19±0.33 | 3.24±0.71 | NA |
|  | Pakchoi cabbage | 0.05±0.04 | 0.02±0.02 | 0.13±0.08 | NA | 0.05±0.05 | 0.54±0.09 | 3.75±2.15 | (0.31±0.53)×10^-1^ |
|  | Leaf lettuce | 0.03±0.03 | 0.01±0.01 | 0.08±0.01 | NA | 0.06±0.02 | 0.56±0.29 | 2.94±1.12 | NA |

**Table S4** Classification grades used in comprehensive pollution index

| Pollution grade | Comprehensive pollution index | Pollution status | Pollution level |
| --- | --- | --- | --- |
| 1 | *NCPI*≤0.7 | Security | Clear |
| 2 | 0.7< *NCPI*≤1.0 | Alert level | Relatively clear |
| 3 | 1.0< *NCPI*≤2.0 | Slight pollution | Suffer pollution |
| 4 | 2.0< *NCPI*≤3.0 | Moderate pollution | Obvious pollution |
| 5 | *NCPI* >3.0 | Heavy pollution | Heavy pollution |

**Table S5** *F* and *p-value of* One-Way ANOVA used to test the differences in the contents of soil properties, eight trace elements in soil and crop among three farmlands

| Soil trace elements | ***F*** | ***p-value*** |  | Crop trace elements | ***F*** | ***p-value*** |
| --- | --- | --- | --- | --- | --- | --- |
| As | 64.27 | **<0.01** |  | As | 0.56 | >0.05 |
| Cd | 9.58 | **<0.01** |  | Cd | 23.07 | **<0.01** |
| Cr | 8.46 | **<0.01** |  | Cr | 96.23 | **<0.01** |
| Cu | 69.46 | **<0.01** |  | Cu | 0.56 | >0.05 |
| Hg | 113.15 | **<0.01** |  | Hg | 57.59 | **<0.01** |
| Ni | 1.47 | >0.05 |  | Ni | 16.19 | **<0.01** |
| Pb | 15.61 | **<0.01** |  | Pb | 5.54 | **<0.01** |
| Zn | 40.12 | **<0.01** |  | Zn | 489.83 | **<0.01** |
| pH | 6.84 | **<0.01** | | | | |
| CEC | 155.01 | **<0.01** | | | | |
| SOM | 35.07 | **<0.01** | | | | |

**Table S6** Risk Control Standard for soil trace element contamination of agricultural land (GB 15618-2018). Bold numbers are the selected standard of soil trace element contamination in this study. The reason this was done is due to the fact that the pH of all sampled points ranged from 6.80 to 14.30, with the mean ± SD of 8.91±2.83. All of the sampled farmlands in the Xinjiang Uygur Autonomous Region were not paddy because the average annual precipitation was about 170 mm, and the climate was a typical continental climate. In order to maximise the assessment of soil pollution levels, the smaller value of the standard at 6.5<*p*H≤7.5 and at *p*H>7.5 was selected for each trace element as the standard of soil trace element contamination in this study.

| Soil trace elements | Type of agricultural land | Risk control guideline (mg·kg^-1^) | | | |
| --- | --- | --- | --- | --- | --- |
|  |  | *p*H≤5.5 | 5.5<*p*H≤6.5 | 6.5<*p*H≤7.5 | *p*H>7.5 |
| As | Paddy felid | 30.00 | 30.00 | 25.00 | 20.00 |
|  | Non-paddy field | 40.00 | 40.00 | 30.00 | **25.00** |
| Cd | Paddy felid | 0.30 | 0.40 | 0.60 | 0.80 |
|  | Non-paddy field | 0.30 | 0.30 | **0.30** | 0.60 |
| Cr | Paddy felid | 250.00 | 250.00 | 300.00 | 350.00 |
|  | Non-paddy field | 150.00 | 150.00 | **200.00** | 250.00 |
| Cu | Orchard | 150.00 | 150.00 | 200.00 | 200.00 |
|  | Grain and vegetable fields | 50.00 | 50.00 | **100.00** | 100.00 |
| Hg | Paddy felid | 0.50 | 0.50 | 0.60 | 1.00 |
|  | Non-paddy field | 1.30 | 1.80 | **2.40** | 3.40 |
| Pb | Paddy felid | 80.00 | 100.00 | 140.00 | 240.00 |
|  | Non-paddy field | 70.00 | 90.00 | **120.00** | 170.00 |
| Ni | All farmlands | 60.00 | 70.00 | **100.00** | 190.00 |
| Zn | All farmlands | 200.00 | 200.00 | **250.00** | 300.00 |

**Table S7** Risk Control Standard of trace elements in crops in China. Risk Control Standard is the minimum harmful content of trace elements in vegetables, grain, melon and fruits to human health, which is regulated by China Health Ministry. China did not published the Ni has no risk control standard of Ni, thus the standard of Fu, Hu and Yu (1999) were used in this study (Fu YG, Hu X and Yu SX. Study on the tolerance limit of Nickel in food. Journals of Zhejiang University, 1999, 37: 9-11).

| Trace elements | Risk Control Standard (mg·kg^-1^) | | | Reference |
| --- | --- | --- | --- | --- |
|  | Grain crops | Vegetables | Melon and fruits |  |
| As | ≤0.70 | ≤0.50 | ≤0.50 | GB 4810-94 |
| Cd | ≤0.05 | ≤0.05 | ≤0.03 | GB 15201-94 |
| Cr | ≤1.00 | ≤0.50 | ≤0.50 | GB 14961-94 |
| Cu | ≤10.00 | ≤10.00 | ≤10.00 | GB 15199-94 |
| Hg | ≤0.02 | ≤0.01 | ≤0.01 | GB 2762-94 |
| Ni | ≤0.40 | ≤0.40 | ≤0.40 | Fu , Hu and Yu (1999) |
| Pb | ≤0.40 | ≤0.20 | ≤0.20 | GB 14935-94 |
| Zn | ≤50.00 | ≤20.00 | ≤5.00 | GB 13106-91 |

**Table S8** The parameters of generalized linear models (GLM) that used to test the relationships of soil trace element contents, soil contamination level (soil NCPI), soil physicochemical properties, plant types and growth time against crop trace element contents.

| Crops | pH | CEC | SOM | Soil As | Soil Cd | Soil Cr | Soil Hg | Soil Pb | Soil Cu | Soil Zn | Soil Ni | Soil NCPI | Plant type | Growth time | *F*-test (*P*-value) | *R*^2^ |
| --- | --- | --- | --- | --- | --- | --- | --- | --- | --- | --- | --- | --- | --- | --- | --- | --- |
| As | **5×10**^−^**^3^***** | **−1×10**^−^**^3^*** | 8×10^−4^ | **1×10**^−^**^3^a** | **0.10***** | 7×10^−4^ | **−0.05*** | −1×10^−3^ | −1×10^−3^ | **7×10**^−^**^4^a** | **−1×10**^−^**^3^*** | −0.02 | **4×10**^−^**^3^a** | −9×10^−4^ | 5.63(***) | 0.13 |
| Cd | **−1×10**^−^**^3^a** | **1×10**^−^**^3^**** | −1×10^−3^ | 2×10^−3^ | −9×10^−4^ | **9×10**^−^**^4^a** | 0.02 | −1×10^−3^ | **−1×10**^−^**^3^a** | −13×10^−4^ | −1×10^−3^ | 3×10^−3^ | **1×10**^−^**^3^a** | **−12×10**^−^**^4^a** | 6.22(***) | 0.14 |
| Cr | **−1×10**^−^**^3^a** | −1×10^−3^ | 7×10^−4^ | 5×10^−3^ | 0.23 | **0.01a** | **−0.03*** | −1×10^−3^ | −5×10^−4^ | **−1×10**^−^**^3^a** | −2×10^−3^ | 0.09 | **−0.13***** | **7×10^−4^ a** | 14.77(***) | 0.28 |
| Hg | −1×10^−3^ | −1×10^−3^ | −1×10^−4^ | −1×10^−3^ | **5×10**^−^**^3^**** | −1×10^−3^ | −3×10^−3^ | −1×10^−3^ | 8×10^−4^ | −13×10^−4^ | **−9×10**^−^**^2^a** | 1×10^−3^ | **−1×10**^−^**^3^**** | **6×10^−4^ a** | 10.92(***) | 0.23 |
| Cu | 0.03 | −7×10^−3^ | **2×10**^−^**^3^a** | **−0.03*** | **−1.33*** | −7E−3 | **1.67**** | **0.02*** | **0.01a** | **−0.01***** | **0.02a** | **0.72*** | **−0.18*** | 8×10^−4^ | 3.93(***) | 0.10 |
| Pb | **−4×10**^−^**^3^*** | **2×10**^−^**^3^***** | 1×10^−3^ | −1×10^−3^ | 0.05 | 6×10^−4^ | 0.02 | **2×10**^−^**^3^***** | −13×10^−4^ | **−9×10**^−^**^2^**** | **−1×10**^−^**^3^a** | −5×10^−3^ | **0.02**** | **9×10**^−^**^4^***** | 7.45(***) | 0.17 |
| Zn | **−0.21*** | **0.12***** | −1×10^−3^ | **−0.27***** | 2.80 | **−0.11***** | **8.88***** | −0.05 | 0.02 | **6×10**^−^**^3^a** | **0.19***** | **9.92***** | **−4.94***** | **−0.02***** | 46.98(***) | 0.56 |
| Ni | −2×10^−3^ | **−1×10**^−^**^3^***** | −1×10^−3^ | **4×10**^−^**^3^***** | 0.02 | **−1×10**^−^**^3^**** | −0.01 | **−1×10**^−^**^3^*** | −12×10^−4^ | **8×10**^−^**^4^**** | **4×10**^−^**^3^***** | **−0.10***** | **−5×10**^−^**^3^ a** | −7×10^−4^ | 6.44(***) | 0.15 |

NOTE: *p* <0.001 ***; *p* <0.01**; *p* <0.05*; *p* <0.1a.

**Fig. S1** A descriptive map of 535 sampling sites in Xinjiang Uygur Autonomous Regions. The vector map of Xinjiang is downloaded from The Gateway to Astronaut Photography of Earth website (<https://eol.jsc.nasa.govSearchPhotos/>). Because the downloaded maps from this website are free and open to scholars around the world, our study do not need to supply a copyright statement.

**
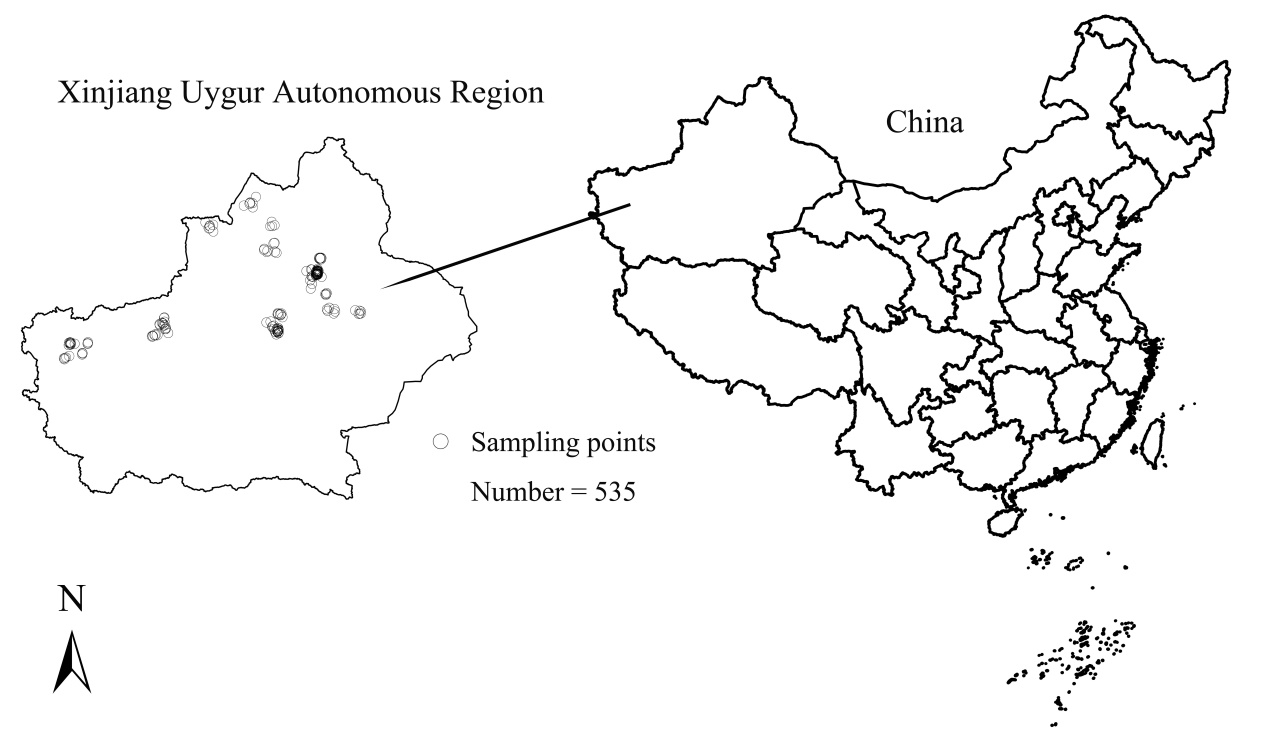
**

**Fig. S2** Difference in enrichment coefficient of eight trace elements among three types of crops.


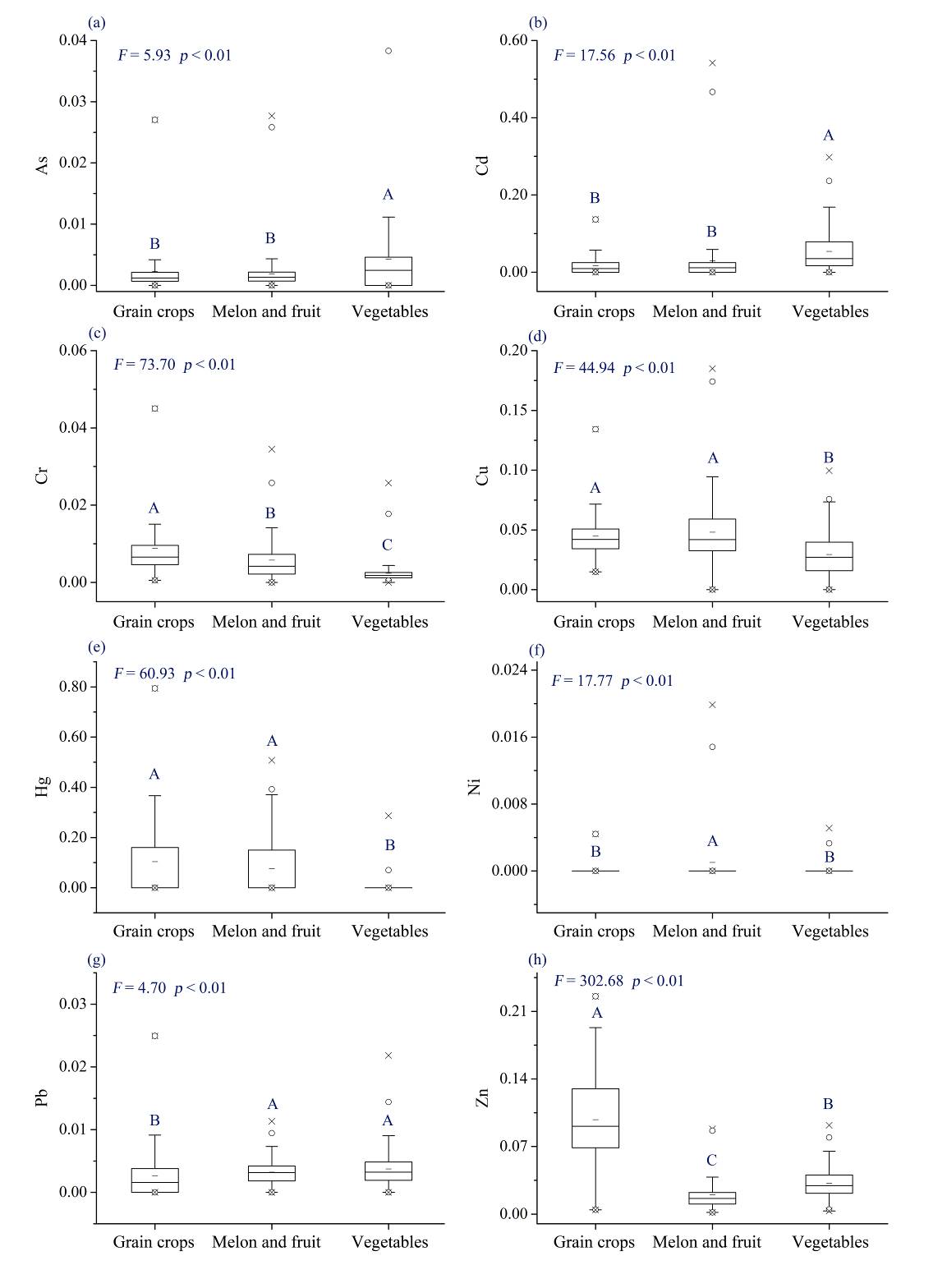

Supplement: Supplementary file 1 — Supplementary Information [file 41598_2021_81764_MOESM1_ESM.docx]
